# Shrinking Wings for Ultrasonic Pitch Production: Hyperintense Ultra-Short-Wavelength Calls in a New Genus of Neotropical Katydids (Orthoptera: Tettigoniidae)

**DOI:** 10.1371/journal.pone.0098708

**Published:** 2014-06-05

**Authors:** Fabio A. Sarria-S, Glenn K. Morris, James F. C. Windmill, Joseph Jackson, Fernando Montealegre-Z

**Affiliations:** 1 School of Life Sciences, Riseholme Campus, University of Lincoln, Lincoln, Lincolnshire, United Kingdom; 2 Department of Biology, University of Toronto at Mississauga, Mississauga, Ontario, Canada; 3 Centre for Ultrasonic Engineering, Department of Electronic and Electrical Engineering, University of Strathclyde, Glasgow, United Kingdom; University of Arkansas, United States of America

## Abstract

This article reports the discovery of a new genus and three species of predaceous katydid (Insecta: Orthoptera) from Colombia and Ecuador in which males produce the highest frequency ultrasonic calling songs so far recorded from an arthropod. Male katydids sing by rubbing their wings together to attract distant females. Their song frequencies usually range from audio (5 kHz) to low ultrasonic (30 kHz). However, males of *Supersonus* spp. call females at 115 kHz, 125 kHz, and 150 kHz. Exceeding the human hearing range (50 Hz–20 kHz) by an order of magnitude, these insects also emit their ultrasound at unusually elevated sound pressure levels (SPL). In all three species these calls exceed 110 dB SPL rms re 20 µPa (at 15 cm). Males of *Supersonus* spp. have unusually reduced forewings (<0.5 mm^2^). Only the right wing radiates appreciable sound, the left bears the file and does not show a particular resonance. In contrast to most katydids, males of *Supersonus* spp. position and move their wings during sound production so that the concave aspect of the right wing, underlain by the insect dorsum, forms a contained cavity with sharp resonance. The observed high SPL at extreme carrier frequencies can be explained by wing anatomy, a resonant cavity with a membrane, and cuticle deformation.

## Introduction

Various animal taxa use ultrasound (>20 kHz) from bats and cetaceans to insects. They use these shorter wavelengths for orientation and communication with mates and rivals [1]. Among insects, orthopterans are known for calling acoustically with many species sensitive to ultrasound [2]–[6]. Tettigoniidae (katydids) are exceptional Orthoptera in the extent to which they exploit ultrasound. Based on a survey of published sound recordings that address the presence of ultrasound, 70% of katydids call using carrier frequencies (carrier in the sense of the most intense spectral peak) beyond 20 kHz [7]. Only about 5% call below 10 kHz, and 25% between 10 and 19 kHz, [1], [7]–[12].

Mapping carrier frequencies on to a katydid phylogeny [13] suggested that ultrasound occurs randomly in species across subfamilies, and is not particularly associated with broadband or pure-tone calls. A large number of neotropical species however, use pure-tone calls in the ultrasonic range [8], [14]–[18], commonly exploiting frequencies in the range 20–45 kHz, identified here as moderately ultrasonic. A few species communicate in the high-ultrasonic range 50–90 kHz, whereas extreme ultrasonic calls (>100 kHz) are rare [7], [19]. Some species communicating in the high and extreme ultrasonic ranges are shown in Table S1.

Montealegre-Z et al. [20] describe the biomechanics of stridulation in katydids that use extreme ultrasound. These authors report a species of katydid identified as *Arachnoscelis* sp. from Colombia, with an unusual ultrasonic call for sound communication consisting of a narrow-band tone at 128 kHz. At the time this constituted the highest ultrasonic mating call (as dominant carrier) ever recorded for an arthropod. That article was written by two of the authors of the present paper (FM-Z, GKM), and we erroneously identified two of the species described herein (*S. aequoreus* and *S. piercei*) within the genus *Arachnoscelis*, under a single species *Arachnoscelis* sp. As such, the carrier frequency value given (128 kHz) in 2006 [20] was actually the average of the two species (1 male of each species).

In this article we describe a new genus *Supersonus* and incorporate three new species within it: *S. aequoreus, S. piercei*, and *S. undulus*. The creation of this genus is required as these insects cannot be assigned to the genus *Arachnoscelis* as recently shown by Montealegre-Z et al. [21].

Males of these species produce unusually high ultrasonic mating calls, and here we report that male *S. aequoreus* emits the highest ultrasound calling carrier ever recorded in nature: 150 kHz. Using Laser Doppler Vibrometry (LDV) and high-speed video (HSV) we demonstrate that the observed extreme frequencies are produced by tiny tuned sound generator, the right wing, which approaches a monopole sound source in its efficient emission of loud ultrasonic signals.

## Material and Methods

### Ethics statement

This study was carried out in strict accordance with the recommendations in the Guide for the Care and Use of Laboratory Animals of the National Institutes of Health. The protocol was approved by the Committee on the Ethics of Animal Experiments of the University of Lincoln, UK (Permit Number: EA1EA2 14/9), and all efforts were made to minimize suffering.

### Field sampling

#### Depositories

The material studied in this project is deposited in the following collections:

MEUV  =  Museo de Entomología Universidad del Valle, Cali, Colombia.

MEUCE  =  Museo de Entomología, Pontificia Universidad Católica del Ecuador, Quito, Ecuador.

MNHN: Muséum National d'Histoire Naturelle, Paris, France

#### Localities

National Natural Park (PNN) Gorgona: PNN Gorgona encompasses the islands of Gorgona and Gorgonilla 35 km from the coast of Colombia (lat 2°47′ to 3°6′ N; long 78°6′, to 78°18′W). The park has an area of 13.33 km^2^ with an elevation above sea level of 338 m. The average annual temperature is 26°C, and annual rainfall is 6891 mm. Ecologically, it is tropical rainforest with similar habitat as on the mainland. The nearest point on the mainland is Punta Reyes where the town of Bazán is located in the municipality of El Charco Nariño.

Watershed Pericos: This site is within the small community of El Salto, of the municipality of Buenaventura, Valle del Cauca (lat. 3° 56′N, long 76° 47′W). The watershed is located at the 76-km point of Route 40, the road that goes from the city of Cali to Buenaventura. Due to its location on the outskirts of the Andean western cordillera, this area is considered tropical rainforest, with rainfall between 4,000 and 10,000 mm per year. Mean temperature fluctuates daily between 18°C and 25°C [22].

Tinalandia: This site is located in a small private forest reserve in the province of Pichincha, (lat 0° 19′ S, long 79° 30′W; 600 m elevation) 112 km southwest of Quito on the road to Santo Domingo de los Colorados and 16 km southeast of Santo Domingo. The vegetation is lowland rainforest, typical of the western slopes of the Andes, with faunal affinities extending into Colombia [23].

### Specimen collection

Males and females of *Supersonus* spp. were initially collected at night by searching understory vegetation using headlamps. However this method proved quite inefficient. After almost 15 years of near fruitless hunting, we discovered that species of this genus inhabit epiphytes such as bromeliads and orchids located between 10-15 m above the ground and a few metres below the canopy (for details of the collecting method Montealegre-Z et al. [24]). Specimens were transported to the University of Lincoln and University of Strathclyde where their songs were recorded using equipment that permitted a wide assessment of the (ultrasonic) sound frequency range.

### Sound recordings

The extreme ultrasonic nature of the calls of *Supersonus* spp. was first reported by Montealegre-Z et al. [20] from one of the species described here, but erroneously identified as *Arachnoscelis* sp. Once aware of the range of sound frequencies exploited by these creatures, we digitized audio recordings at 1200 kilosamples s^−1^. Insects were placed in a mesh cage, and hung from the ceiling of an anechoic room >1 m below the ceiling and >1 m above the floor mitigating against potential reflections. Sound recordings were obtained using a 1/8" Brüel & Kjær Type 4138 condenser microphone (cover not removed), connected to a Brüel & Kjær 2633 preamplifier (Brüel & Kjær, Nærum, Denmark). Data were stored in a notebook computer using an NI USB-6259 board (National Instruments, Austin, TX, USA) and LabVIEW version 9 (32 bit) 2009 software interface (National Instruments, Austin, TX, USA). The microphone's sensitivity was calibrated with a sound-level calibrator (Brüel & Kjær, 4231) and the interface of the Polytec Scanning Vibrometer software (version 8.5; Polytec, Waldbronn, Germany). The sound level calibrator produces a 1 kHz tone at 1.0024 Pa (94 dB). The microphone response was corrected digitally in the Polytec software interface against the calibrator using a correction factor, until a value of 1.0024 Pa was reached in the spectrum of the signal as seen in the analyzer window. The monitoring microphone was placed at 1 cm away, but at the same height to, the specimen. The speaker was placed at 15 cm ventral of this preparation. Signals could be recorded very accurately using this transduction interface.

### Wing resonance measurements

The wings of *Supersonus* spp. are unusually small (<0.4 mm^2^, see Fig. 2), and as such positioning a living insect with its wings extended for LDV scanning and resonance stimulation is challenging. Measurements of wing resonances were achieved only from two males of *S. piercei* and one of *S. undulus*, both species described here. Resonances were excited with periodic chirps including frequencies in the range 5–50 kHz, and 20–200 kHz. Chirps were generated by the PSV 300 internal data acquisition board and lasted 80 ms.

Sound was passed to a loudspeaker (ACR, FT 17H, Horn Tweeter, Fostex, Tokyo, Japan, frequency response 5–50 kHz) or to a custom-built ultrasonic transducer (capacitive membrane, frequency response 50–700 kHz, 30 mm diameter aperture; University of Strathclyde, UK) positioned 15 cm from the specimen to ensure the animal was in the far-field up to 200 kHz.

Vibration velocities of the wings were measured by a microscanning laser Doppler vibrometer (Polytec PSV-300-F; Waldbronn, Germany) with an OFV-056 scanning head, fitted with a close-up attachment. The laser beam on the wing membrane was monitored by live video feed to the vibrometer's controlling computer. No reflective particles were required for measuring wing vibrations. The entire stridulatory field of both tegmina was scanned using 150–800 measurement points.

The spectrum of the stimulus was corrected to give equal energy at all frequencies at 80 dB (re 20 µPa SPL) for each insect. Recordings were obtained at 512 –1000 kilosamples per second. The quality of the stimulus was monitored using a 1/8" condenser microphone Brüel & Kjaer Type 4138, connected to a Brüel & Kjaer 2633 preamplifier (Brüel & Kjaer, Nærum, Denmark). For recordings, an intact specimen was mounted on a Blu-Tack (Bostik Ltd, Leicester, UK) holder using metallic clamps to fix its legs. The left wings were laterally extended by fixing the axillary sclerites with beeswax, while the right wing was left in the normal resting position.

Stridulatory movements were recorded at 3000 frames per second using a high-speed video camera NACHi-DCamII (A504kc, NACImage Technology, Simi Valley, CA, USA). Video and sound data were edited and synchronized using VirtualDub V1.9.11 (http://www.virtualdub.org/) and Adobe Premiere Pro CS4 (Adobe Systems Incorporated, San Jose, CA, USA) and analysed frame by frame using the software ImageJ V1.42 (Wayne Rasband, Research Services Branch, National Institute of Mental Health, Bethesda, MD, USA).

### Nomenclatural Acts

The electronic edition of this article conforms to the requirements of the amended International Code of Zoological Nomenclature, and hence the new names contained herein are available under that Code from the electronic edition of this article. This published work and the nomenclatural acts it contains have been registered in ZooBank, the online registration system for the ICZN. The ZooBank LSIDs (Life Science Identifiers) can be resolved and the associated information viewed through any standard web browser by appending the LSID to the prefix “http://zoobank.org/”. The LSID for this publication is: urn:lsid:zoobank.org:pub:9597BAC4-9C41-4269-BDDD-8F554C992875. The electronic edition of this work was published in a journal with an ISSN, and has been archived and is available from the following digital repositories: PubMed Central and LOCKSS, University of Lincoln UK and University of Strathclyde UK repositories.

## Results

### Systematics and taxa description

Order ORTHOPTERA

Family TETTIGONIIDAE

Subfamily LISTROSCELIDINAE

#### *Supersonus* gen. nov

Sarria-S, Morris, Windmill, Jackson & Montealegre-Z, 2014 urn:lsid:zoobank.org:act:93B0C32C-1AC0-498B-90C3-EB3324B163AB

Etymology: Named for *sonus* L. sound, and *super* L. above, in recognition of the elevated forest singing perches of these insects, the presence of sound energy at frequencies significantly above100 kHz, and in acknowledgement of the original designation of ultrasonic sound wavelengths as ‘supersonic’ (e.g, Pierce [25]).

**Figure 1:**
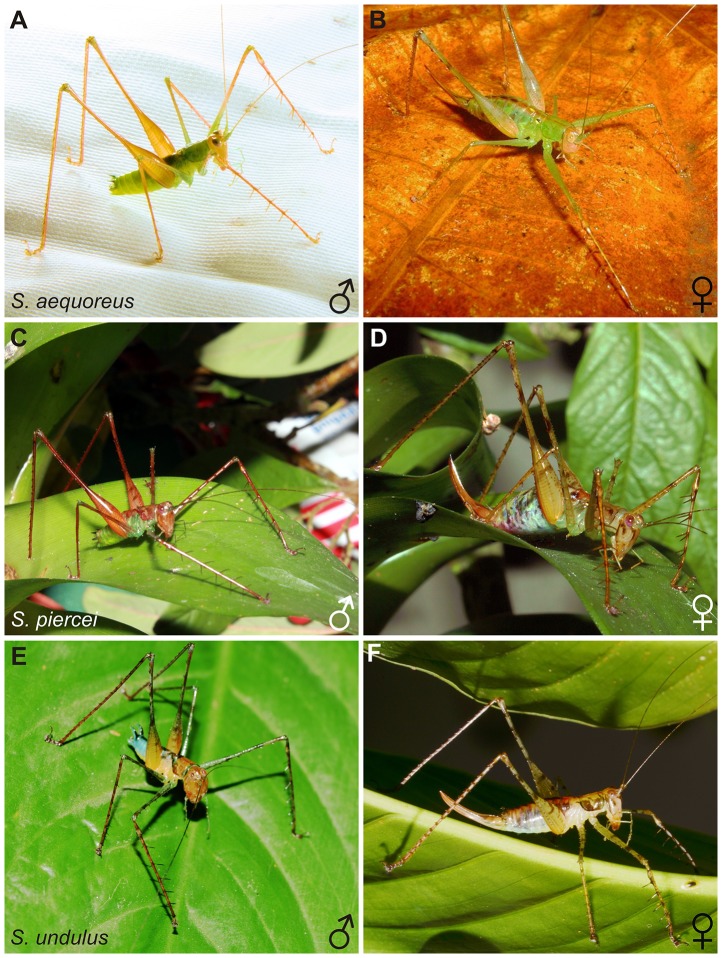
Morphological comparision of *Supersonus* spp. habitus. (A, B) Male and female of *S. aequoreus*. (C, D) Male and female of *S. piercei*, and (E, F) Male and female of *S. undulus*. (A) Under a CC BY license, with permission from Natasha Mhatre, original copyright 2010. (C, D) Under a CC BY license, with permission from Manuel Jara, original copyright 2014. (B, E, F) Under a CC BY license, with permission from Fernando Vargas-Salinas, original copyright 2011.

**Figure 2:**
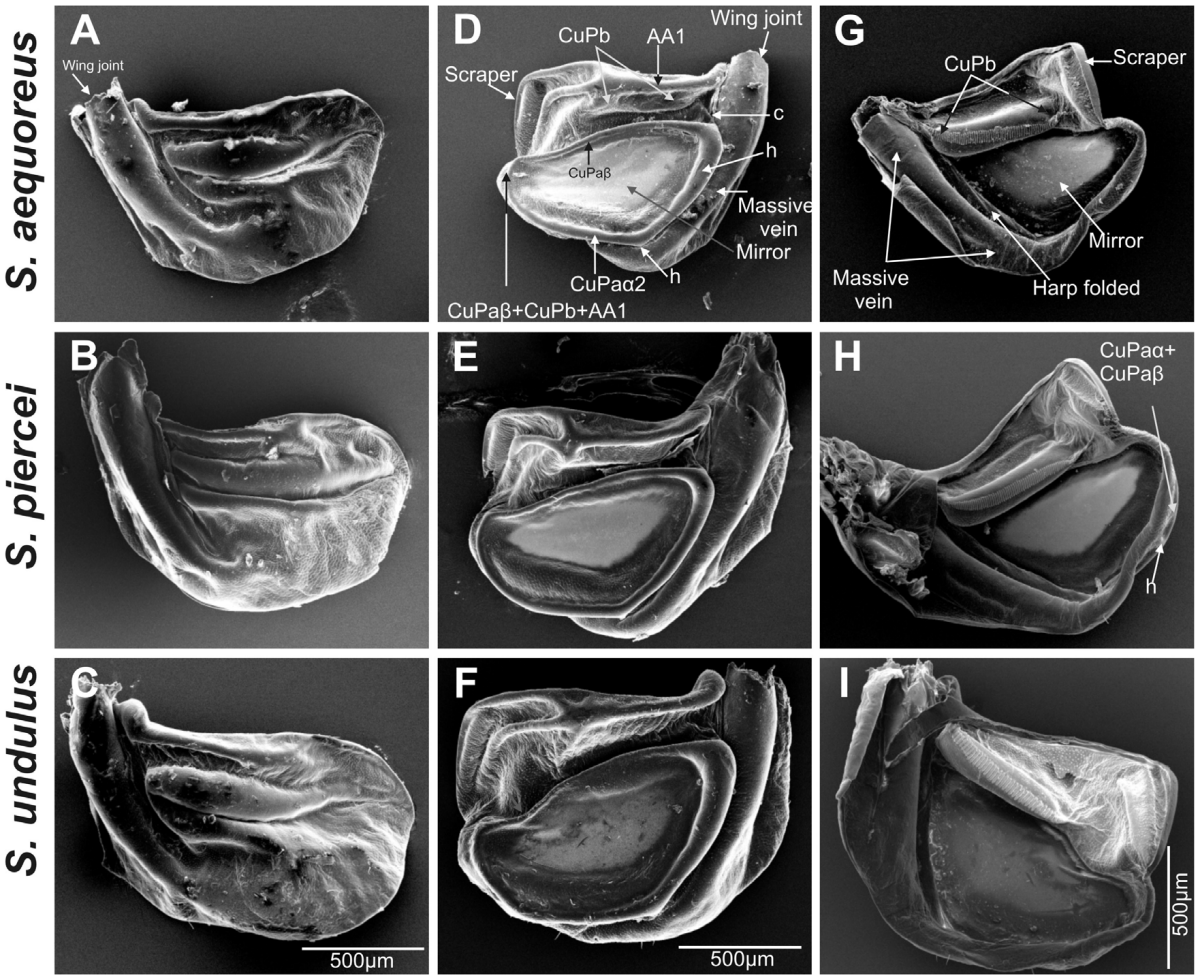
Male wing morphology comparison between *Supersonus* spp. (**A–C**). Left wing dorsal view. (**D–F**) Right wing dorsal view. (**G–I**) Right wing ventral view. First row of images*: S. aequoreus*, middle row: *S. piercei*, and third row: *S. undulus*.

**Figure 3:**
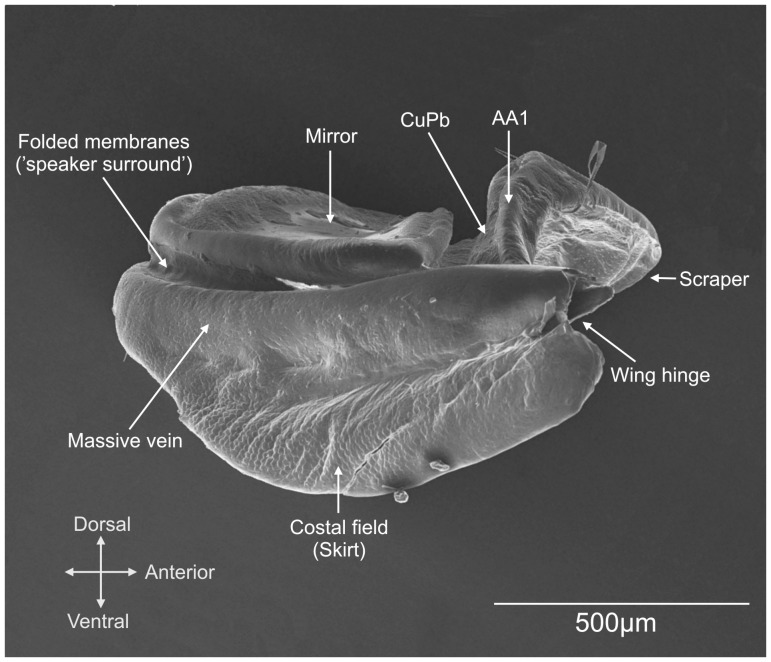
Male right wing morphology of *Supersonus piercei* (right side view).

**Figure 4:**
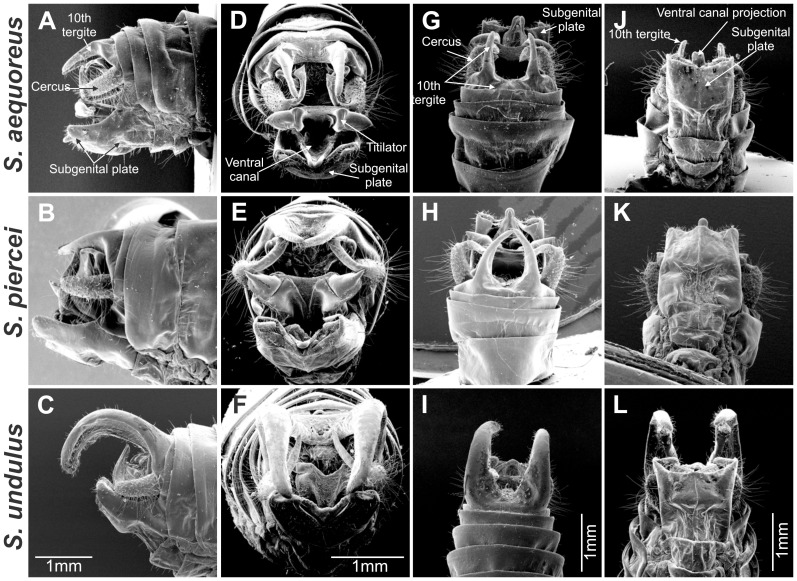
Male genitalia morphology comparison between *Supersonus* spp. (**A–C**) Lateral view. (**D–F**) Frontal view. (**G–I**) Dorsal view. (**J–L**) Ventral view. First row of images: *S. aequoreus*, middle row: *S. piercei*, and third row *S. undulus*.

**Figure 5:**
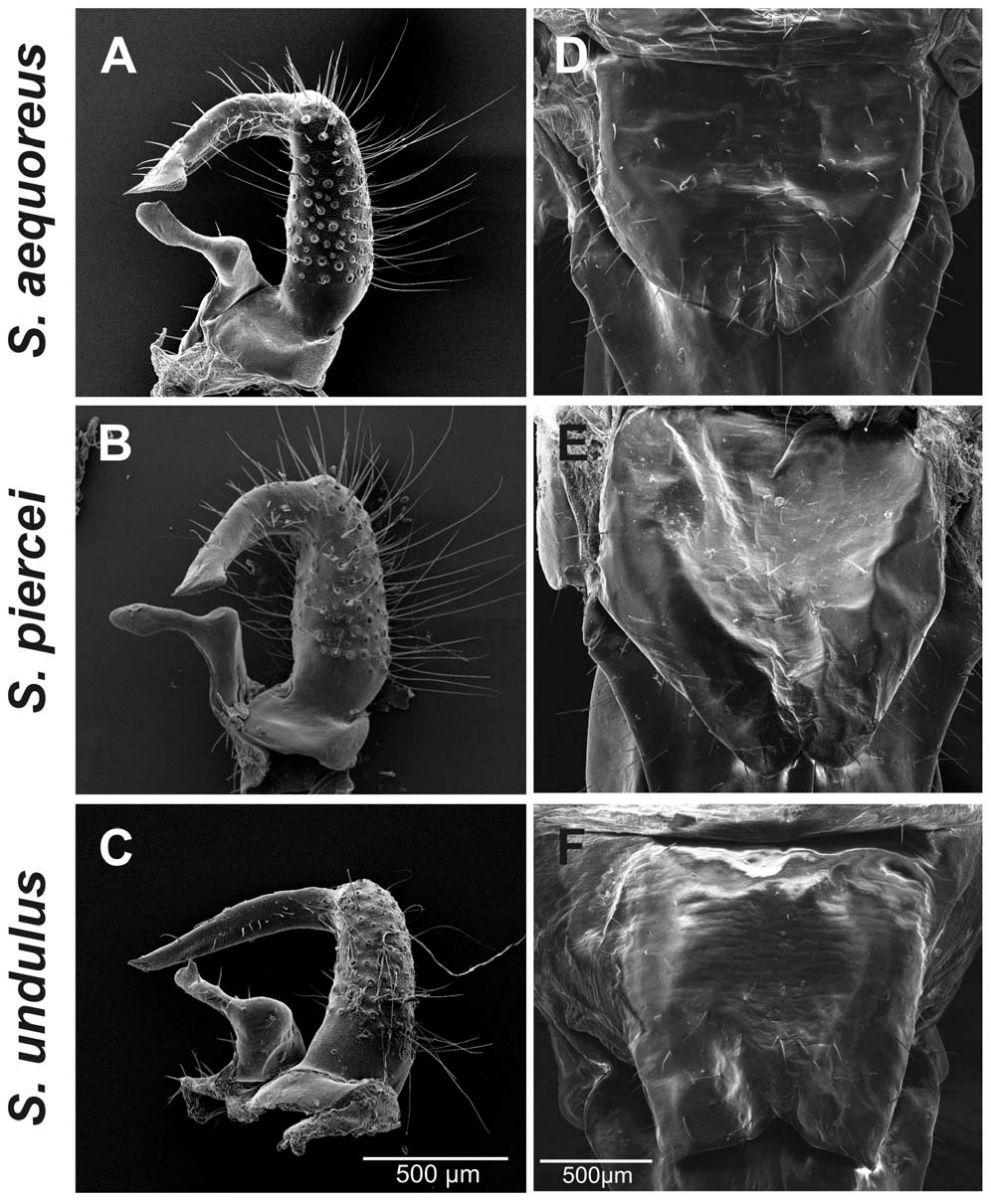
Left male cerci morphology (dorsal aspect) and female subgenital plate comparison across *Supersonus* spp. **(A–C)** Left male cerci. **(D–F)** female subgenital plate.

Diagnosis: Brachypterous insects, hind wings absent in both males and females; forewing (tegminal) sound generators extremely reduced in males to just the size of the stridulatory field (an area of <0.4 mm^2^) and with brief costal region; tegmina reduced to two minute scaly appendages in females (Figs. 1, 2 & 3). Tegmina strongly asymmetrical; right tegmina with subtriangular mirror speculum frame protruding robustly above the tegminal contour and speculum plane (Fig. 3). Tympanal slits asymmetrical, the internal or anterior slit smaller than posterior or external slit (Fig. S1). Cerci inflated in basal half, with a distal narrow extension anteriorly incurved and upturned. Cerci bear a basally articulated appendage, which projects upwards and abruptly bends posterolaterally (Figs. 4 & 5). Titillators present in the form of two sclerotized dentate structures projected laterad; not protruding from the terminalia contour. A sclerotized canal, ventral to the titillators, lies on top of the dorsal face of subgenital plate and slightly protrudes from the plate contour (as seen ventrally and dorsally, Figs. 4J, 4K, & 4L). Male subgenital plate truncate, with two minute movable incurved styles. Female subgenital plate short, usually pentagonal, medially notched and bilobulated. The females exhibit spotted integument (Figs. 1B, 1D & 1F). Egg with micropyle located in a depression on the anterior end (Fig. S2).

#### *Supersonus aequoreus* sp. Nov

Sarria-S, Morris, Windmill, Jackson & Montealegre-Z, 2014 urn:lsid:zoobank.org:act:82E22BAC-E5B5-4A7D-B4DB-04EBB9ED605A.

Etymology: *aequoreus* L., surrounded by the sea. Named as singing from an island environment surrounded by the ocean.

Diagnosis: Species recognized by male cercal morphology, right mirror area, stridulatory file tooth arrangement and call carrier frequency.

**Figure 6:**
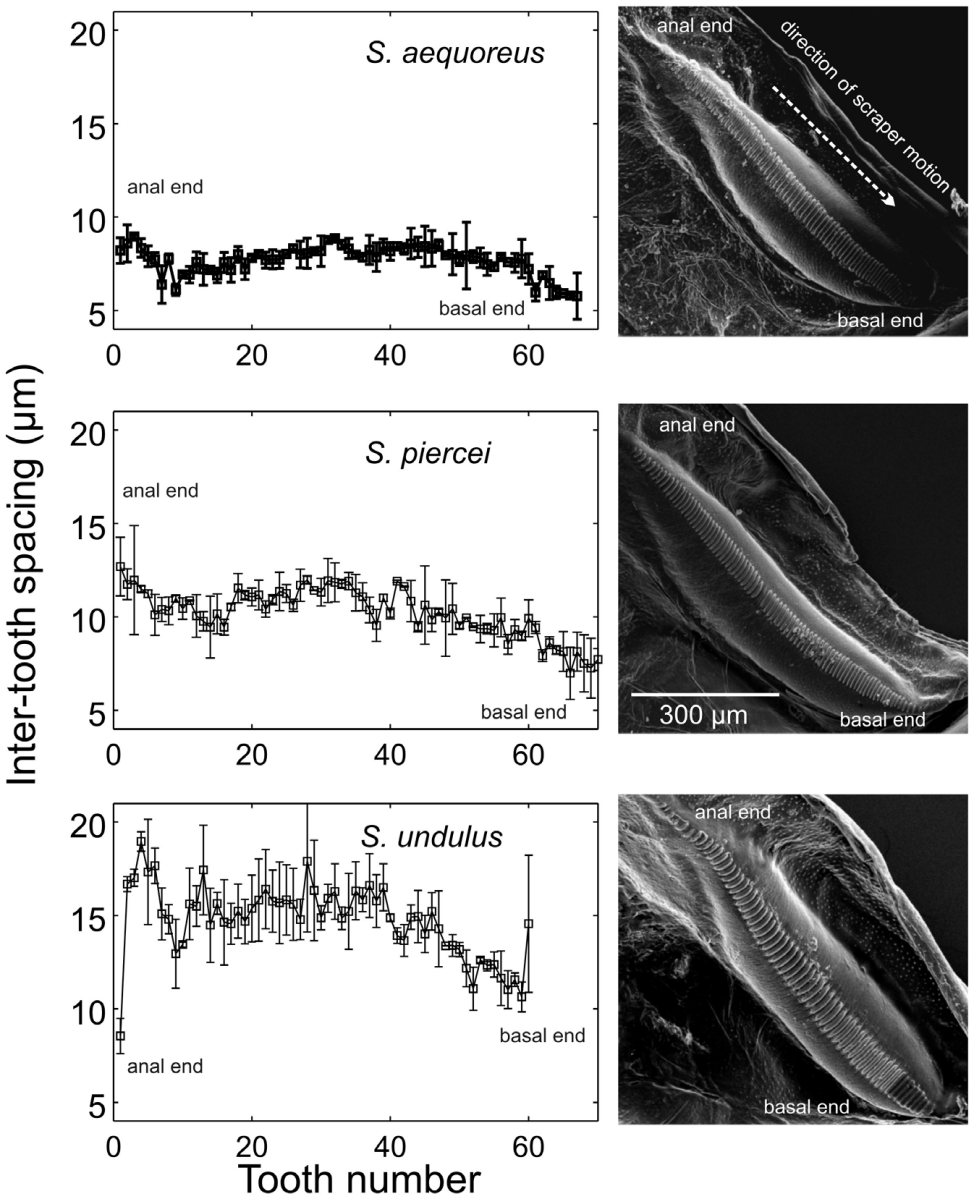
The stridulatory file of *Supersonus* spp. Graphs panels on the left show measurements of inter-tooth distances in the direction of scraper motion during stridulation (anal to basal). Panels on the right show SEM pictures of the files of each species.

Description: Wings– Right speculum area ca 0.22 mm^2^ (Figs. 2D & 2G). Stridulatory file bearing 67–75 teeth. Measured from anal side of the file, inter-tooth spacing varies as shown in Fig. 6, with an average of 7.8 µm (±0.8 µm). Abdomen– Male tenth tergite with two down curved short projections (≤1 mm) separated by a broad shallow notch (Figs. 4A, 4D, & 4G). Titillators two highly sclerotized sclerites bearing apically two randomly organised rows of small teeth, projected laterad (Fig. 4D). Elongated distal half of male cerci with apex laterally expanded, sharply flattened, ventral surface minutely spiculate. Articulated cercus appendage dorso-posteriorly projected, with proximal half dorsally flattened; distal half bent internally, spatulate, with pre-apical dorsal tooth (Fig. 5A). Male subgenital plate basally expanded, nearly truncate, bearing two minute lateral styli which project inwards (Fig. 4D & 4J). Female subgenital plate distally rounded, with a minute medial notch (Fig. 5). See Table 1 for anatomical measurements.

**Table 1:** Measurements (in mm) of some morphological structures of *Supersonus* spp. F =  fore, M =  mid, H =  hind, S =  subgenital.

		*S. aequoreus*				*S. piercei*			*S. undulus*			
		Male (n = 2)		Female (n = 2)		Male (n = 2)		Female (n = 1)	Male (n = 3)		Female (n = 2)	
		Mean	SDV	Mean	SDV	Mean	SDV		Mean	SDV	Mean	SDV
Body		10.55	0.21	12.8	0.28	12.75	0.35	11.5	13.12	0.27	18.04	0.25
Pronotum		3	0	3.2	0	3.2	0	3.5	2.9	0.07	3.36	0.03
Mirror area		0.23	-	-	-	0.32	0	-	0.4	-	-	-
File length		0.73	-	-	-	0.84	0	-	0.84	-	-	-
F-femur		13.45	0.07	10.15	0.07	14	0	11.5	12.31	0.04	10.68	0.42
M-femur		10.55	0.21	8.1	0	10.95	0.49	9.2	10.56	1.33	9.18	0.18
H-femur		18.9	0.14	18.9	0.56	20.4	0	20.8	19	0.48	19.74	0.02
F-tibia		15.3	0.42	11.6	0.14	15.5	0	13.4	15.24	1.21	12.45	0.06
M-tibia		12.15	0.35	9.25	0.35	14.4	3.25	10.5	12.28	1.43	10.23	1.03
H-tibia		19.7	0.14	18.8	0.92	20.65	0.49	21.4	19.85	0.47	20.68	0.14
S-Plate	Length	1.52	0.11	1.15	0.07	1.62	0.11	1.6	1.35	0.07	1.48	0.03
	Wide	1.16	0.06	1.6	0.14	1.61	0.27	1.5	1.35	0.07	1.44	0.07
Cercus		1.77	-	1.1	0.14	1.63	-	1.3	1.68	-	1.1	-
Ovipositor		-	-	9.6	0.21	-	-	10	-	-	10.56	0.87

Coloration: Sexual dimorphism is observed in the coloration pattern (Figs. 1A & 1B). Males: Head, hind legs, first and middle tibiae fulvous (Fig. 1A). Facial marks absent. Pronotum and abdomen smaragdine. Wings olivaceous. Tenth tergite projections dorsally fuscous. Females: Female body coloration varies from smaragdine to resinous-amber, with a longitudinal fuscous strip on tergum (Fig. 1B). Femur and tibia fulvotestaceous, with mottled patterns. Head fulvous with two brunneus vertical lines on occiput, rostrum with two vertical brunneus lines delineating the eye sockets. Subgenital plate and cerci amber with a fuscous spot on the distal section, ovipositor fulvotestaceous.

Material examined: Holotype: 1♂, Colombia, Cauca, Guapi, PNN Gorgona. November, 2009, (F. Sarria-S). Allotype: 1♀; May 17, 2007 (F. Sarria-S). 1♀, Colombia, Cauca, Guapi, PNN Gorgona. November 16, 2007, (F. Montealegre-Z). Paratypes: 4, Colombia, Cauca, Guapi, PNN Gorgona. 1♀, December 14, 2003, (G. K. Morris). 1♂; December 19, 2003 (F. Montealegre-Z). 1♀; November 16, 2007, (F. Montealegre-Z). 1♂; November 16, 2007 (F. Montealegre-Z). Depository: All material deposited at MEUV.

#### *Supersonus piercei* sp. Nov

Sarria-S, Morris, Windmill, Jackson & Montealegre-Z, 2014 urn:lsid:zoobank.org:act:F9E06E3B-41F0-4193-B5FA-0D184DF4258C.

Etymology: Species named in recognition of George W. Pierce, Professor of Physics, Harvard Univ., progenitor of ultrasound study, who wrote in his classic 1948 ‘The songs of Insects’: “…these researches show that frequencies extending from the audible to the superaudible exist in the sounds emitted by insects. With some classes of insects I have found that the significant part of their sounds is of a frequency so high as to be entirely inaudible to the human ear…” [25].

Diagnosis: Species recognized by male genitalia, right mirror area, stridulatory file tooth arrangement, and call carrier frequency.

Distribution: Continental rain forests of the biogeographic Chocó, situated within Valle del Cauca, Colombia. Specimens have been collected from Bajo Anchicayá, Bajo Calima, and near the Pacific coast at Papayal.

Wings - Right mirror area ca 0.31 mm^2^ (Figs. 2E and 2H). Stridulatory file bearing 72–84 teeth. Measured from the anal side of the file, inter-tooth spacing varies as shown in Fig. 6, and average tooth pitch is 10.3 µm (±1.5 µm). Abdomen - Male tenth tergite bearing two incurved elongate projections with nearly converging tips; basally these projections separated by a broad shallow notch (Figs. 4E & 4H). Titillators heavily sclerotized, acute, projected laterally (Fig. 4E). Male cerci basally broad, incurved, distal half elongated, upturned, with tip flattened and laterally expanded (Figs. 4E & 4H). Male subgenital plate distally undulate, bearing two minute styli projected inwards and hardly differentiated from the distal plate contour (Figs. 4E, 4H & 4K). Female subgenital plate subtriangular, with a V-shaped notch (Fig. 5E). See Table 1 for anatomical measurements.

Coloration: Coloration pattern sexually dimorphic (Figs. 1C & 1D). Male coloration: Head and limbs fusco-ferruginous (Fig. 1C). Facial marks present. Pronotum and tergites of thorax fusco-ferruginous. Abdomen smaragdine with a median fusco-ferruginous strip on tergum. Wings fusco-ferruginous with fuscous margins. Tenth tergite projections dorsally fuscous.

Female coloration: Female body caesious with a longitudinal fusco-testaceous strip on tergum (Fig. 1D). Facial marks present. Pronotal disk amber, pronotal lobes irregularly covered with a brunneus-atrous macula. Limbs resinous-amber with irregular suffusions of brunneus dots and spots. Subgenital plate and cerci amber, ovipositor fulvous.

Material examined: Holotype: 1♂, Colombia, Valle del Cauca, Buenaventura, El salto, Pericos watershed. September 7, 2013 (F. Sarria-S), MEUV. Allotype: 1♀ Colombia, Valle del Cauca, Buenaventura, Bajo Anchicayá. February 23, 2010 (F. Sarria-S), MEUV. Paratypes: 1♂, Colombia, Valle del Cauca, Buenaventura, El salto, Pericos watershed. September 7, 2011 (F. Sarria-S), MEUV. 1♀ Colombia, Valle del Cauca, Buenaventura, Bajo Anchicayá (Fabio Sarria), MEUV. 1♀ Colombia, Valle del Cauca, Buenaventura, Ladrilleros, 1989 (K. Riede) MNHN, Paris.

#### *Supersonus undulus* sp. Nov

Sarria-S, Morris, Windmill, Jackson & Montealegre-Z, 2014 urn:lsid:zoobank.org:act:922C238F-C587-4B88-8D75-3FAC169A9FF8.

Etymology: *undulus* L. diminutive of wave; named for the shortness of this species' dominant acoustic wavelength (∼2.7 mm).

Diagnosis: Species recognized by male coloration pattern, right mirror area, course of the CuPaβ vein and female subgenital plate.

Description: Wings - Mirror area ca 0.39 mm^2^ (Figs. 2F & 2I), CuPaβ abruptly sinuated near the joint CuPaβ+CuPb+AA1 (Fig. 2F, red arrow). Stridulatory file bearing 60 teeth. Measured from the anal side of the file, inter-tooth spacing varies as shown in Fig. 6, with a tooth pitch average of 13.9 µm (±1.8 µm). Abdomen - Male tenth tergite bearing two elongated projections strongly deflected and separated by a broad U-shape shallow notch (Figs. 4F & 4I). Titillators smaller than in the other two species, apex heavily sclerotized, folded upwards and anteriorly, with serrate margins (Fig. 4F). Ventral canal of titillator rounded and dentate; elongate incurved distal half of male cerci with tip ending acute and slightly curved posteriorly (Fig. 5C). Articulated appendage dorsoposteriorly projected, with proximal half slightly concavely depressed; distal half bent internally, continuously tubular, not flattened, with strong pre-apical dorsal spike (Fig. 5C). Male subgenital plate distally subsinuate, somewhat truncate, with two minute styli projected inward; relative to subgenital plate size, styli are larger than in the other two species (Fig. 4L). Female subgenital plate quadrangular with a V-shaped notch (Fig. 5F). See Table 1 for anatomical measurements.

Coloration: sexually dimorphic (Figs. 1E & 1F). Male coloration: Head fusco-testaceous without facial marks. Tibiae fusco-ferruginous. Anterior and middle femora caesious with brunneus spots. Hind femora proximally fulvous and distally caecious (Fig. 1E). Pronotal disk fusco-testaceous, bearing in the middle a narrow fusco-ferruginous line. Anterior and posterior edges of the pronotal disk olivaceous. Abdominal tergites callainus with mid longitudinal fuscous band, which extends down to apex of the tenth tergite lobules. Thoracic pleura and abdominal sternites sulphureous. Wings fuscous.

Female coloration: Head albus-argentum (pearly) with brunneous marks. Body ventrally albus-argemtum, tergum fulvous with a tranverse fuscescent band on the posterior edges of each abdominal tergite. Longitudinal medial fusco-testaceous line on tergum. Pronotal disk olivaceous, pronotal lobes irregularly covered with a fuscescent macula. Femora olivaceous with irregular suffusions of fuscescent dots and spots. Tibiae fusco-testaceous. Subgenital plate and cerci amber, ovipositor fuscescent.

Material examined: Holotype: 1♂, Ecuador,Pichincha, santo Domingo de los Colorados, Tinalandia; December 18, 2011 (F. Sarria-S & S. Valdés-R), MEUCE. Allotype: 1♀, Ecuador,Pichincha, santo Domingo de los Colorados, Tinalandia; December 18, 2011 (F. Sarria-S & S. Valdés-R), MEUCE. Paratypes: 1♀ Ecuador,Pichincha, Santo Domingo de los Colorados, Tinalandia; July 11–13, 2003 (G.K. Morris, P. Wall, D. Klimas, F. Montealegre-Z). 1♂, same locality as previous, December 15, 2011 (F. Sarria-S & S. Valdés-R). All paratypes deposited at MEUV.

### Acoustics and biomechanics

Measurements of acoustic parameters for all species are shown in Table 2.

**Table 2:** Acoustical and biomechanical measurements of *Supersonus* spp., mean values (refer to text for standard errors).

	Frequency (KHz)	SPL (dB SPL rms re 20 µPa, at 15 cm)	Train duration (ms)	Pulses per train	Oscillations per pulse	Discrete pulse duration	Inter-tooth spacing(µm)
*Supersonus aequoreus*	148.3	115–108	13–19	12.2	8–10	62.91	7.8
*Supersonus piercei*	124.5	111–99	10–28	6.4	7	105	10.3
*Supersonus undulus*	115.21	113.32–103.51	11–21	7.0	6–10	82.1	13.9

#### *Supersonus aequoreus*

**Figure 7:**
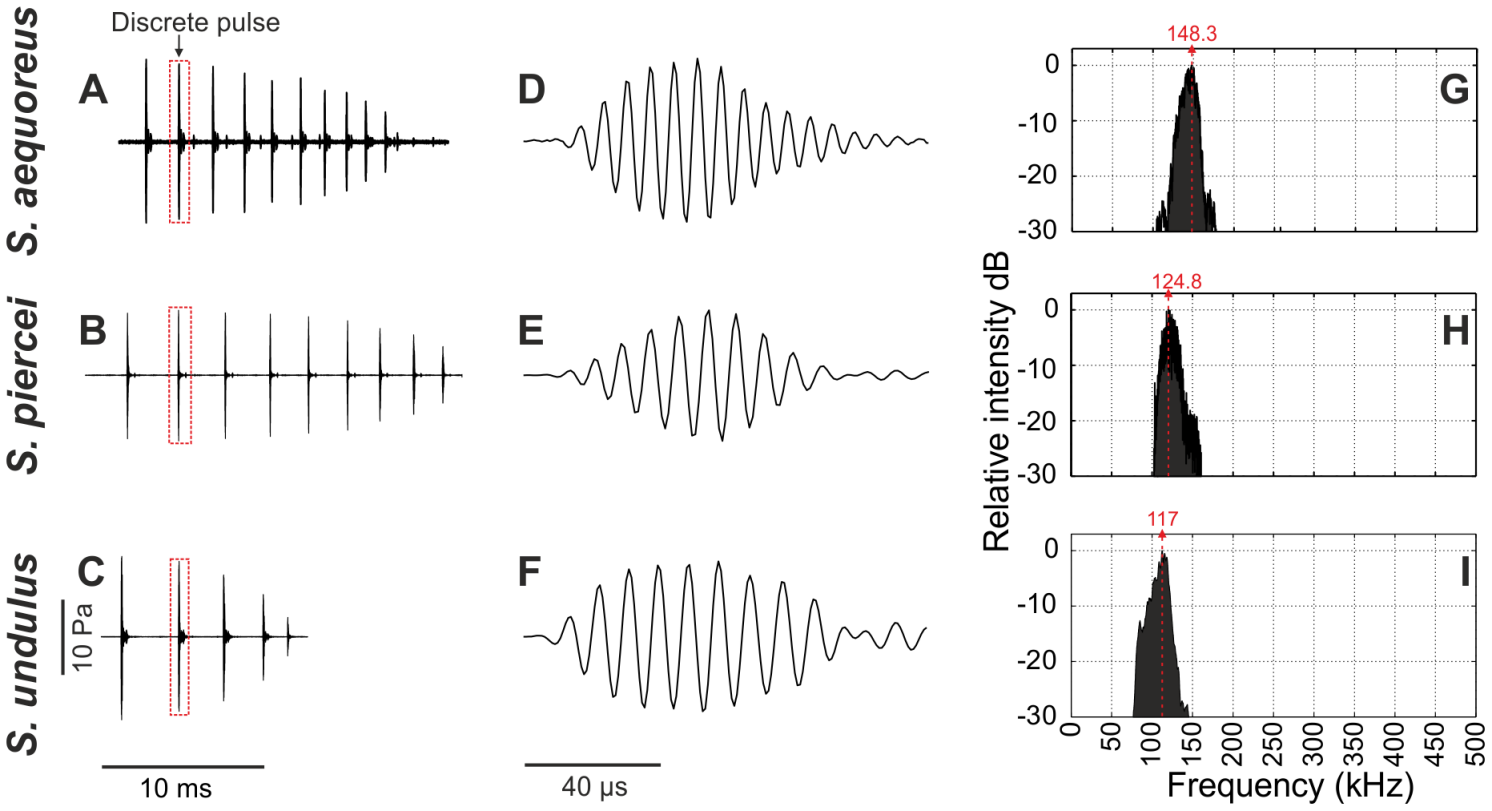
Calling song features of *Supersonus* spp. (**A–C**) Syllable or pulse train produced during a closing stroke of wing motion. (**D–F**). Wave form of a discrete pulse (red rectangle in A, B, and C). (**G–I**). Power spectrum.

Data presented are of five males recorded under laboratory controlled conditions. Each call of *S. aequoreus* is a train of pulses lasting 13–19 ms. In the train are 10 to 13 (average 12.2±1.23, n = 5 males) very brief sinusoidal temporally well-separated pulses (Fig. 7A). In a bout of singing lasting several minutes, these trains occur in groups of two, and each group (call) is repeated every 1.2–1.7 s (as recorded at 24°C). The pulse train period within the group varies between 73 and 80 ms.

Peak amplitudes of early pulses are usually the same for the first 3–4 pulses of the train, gradually decreasing for later ones (Fig.7A) and in mid-train some successive pulses may establish the same peak amplitude peaks. Measured from the start to the end of the train, sound pressure levels vary between 115 and 108 dB SPL rms re 20 µPa (at 15 cm). Each pulse lasts about 62.91 (±4.94) µs (Fig. 7D); pulse period was 1.55±0.31 ms. This implies that discrete pulses are separated by silent intervals of 1.440 (±0.303) –1.480 (±0.301) ms. However, pulse periods are not constant across the train, but gradually decrease from 1.93 to 0.92 ms suggesting the pulse rate gradually increases during the closing phase of the wings. There are 8–10 oscillations in each pulse, the first 6 of which are probably driven oscillations before decay begins. Pulses recur at an average rate of 711/s±60.88. Output energy of the four specimens recorded was centred at 148.3 kHz (range 147.13–151.10 kHz) (Fig. 7G). There was no significant energy below 100 kHz in the spectrum of either male of this species.

Sound is produced by males during the closing phase of the wings (see Video S1). For a frequency of 148 kHz, and an average inter-tooth spacing of 7.8 µm (see Fig. 6), wings are expected to close at an average speed of 1154.40 mm/s. However, HSV recording clearly shows the wings closing with an average speed of only 12.8±1.4 mm/s (n = 2 males). This suggests the scraper is uncoupled from and moving faster than the observed wing motion to provide the necessary high tooth-strike-rate.

Two of the authors (FMZ and GKM) showed in 2006 [20] that for katydids singing below 40 kHz scraper speed was coupled to the instantaneous speed of the entire wing, i.e. the scraper always moves in concert with the wing. The maximum wing speed measured in coupled singers was ca 250 mm/s. Species singing above 40 kHz cannot contract their muscles any faster to gain speed. In the case of *Supersonus* the required speeds would exceed 1000 mm/s. So, these insects uncouple scraper speed from wing speed where the scraper lodges behind a file tooth, deforms to release, then springs forward at the higher speed across a small set of file teeth. Deformation energy thus becomes the basis for enhanced tooth-contact speed.

#### *Supersonus piercei*

Data is presented from four males recorded under laboratory conditions at 24°C. Each syllable of *S. piercei* is again a train of time-discrete pulses, the train lasting 10 to 28 ms (n = 4 males). A train carries between 5 to 13 (6.4±3) of these very brief sinusoidal pulses (Fig. 7B). In a bout of singing lasting several minutes, trains are given in groups of two to five, with a train period of 32–64 ms (n = 4).

Peak amplitudes achieved in early pulses slightly incremented, diminishing steadily in later ones (Fig.7B), and in mid-train a number of pulses in succession may peak uniformly. Each pulse lasts about 105 µs (Fig. 7E) and pulse period varied very little, remaining between 2.39 and 2.42 ms for the three specimens recorded. There were about 7 oscillations in each pulse (apparently driven oscillations) before decay began and about 12 waves including the free decay. Pulses recurred at an average rate of 480/s. Output energy in the 1996 specimen was centred at 124.8 kHz, with 126.5 and 122.2 kHz in each of the specimens collected in 2013 respectively, for an average of 124.5±2.17 kHz (n = 4, Fig. 7H). There was no significant energy below 100 kHz in the spectrum of either specimen measured in 2013. The sound pressure level across each pulse train varied between 111 and 99 dB SPL rms re 20 µPa (at 15 cm).

Sound is produced by males during the closing phase of the wings (see Video S2). For a frequency of 124.5 kHz, and an average inter-tooth spacing of 10.3 µm (see Fig. 6), wings are expected to close at an average speed of 1282.4 mm/s. HSV recording indicates that for sound production the wings in males of this species actually close with an average speed of only 14.6±1.4 mm/s (n = 1 male). Therefore, there is a mismatch between scraper speed and wing speed (see above).

#### *Supersonus undulus*

The data presented correspond to three males recorded at 23°C under lab conditions. Each syllable was a train of discrete pulses lasting 11 to 21 ms. Each train consisted of 5–9 very brief sinusoidal time-discrete pulses each lasting 82.1±12.2 µs (n = 2 males, Fig. 7C). Males interact acoustically in their call timing and after short resting periods. The call of one male stimulates singing in others. Trains are produced individually with a period of 204±64.2 ms (n = 2). The average number of pulses in the train was 7.0±2 (n = 2).

As in the other two species, peak amplitude was usually high in the initial pulses, and gradually decreasing in later ones (Fig. 7C). During the first half of the train, a series of pulses sometimes showed uniformly high amplitudes. The sound pressure level across pulses in each pulse train varied with these amplitudes between 113.32 and 103.51 dB SPL rms re 20 µPa (at 15 cm). The pulse period decreased gradually from 2.93 to 1.52 ms for the three specimens recorded. The number of waves in each pulse (driven oscillations) before decay varied between 6 and 10 (Fig. 7F). The pulses were produced at an average rate of 441.76/s±23.87. Output energy was centred at 115.21±4.40 (n = 2, Fig. 7I). Spectral energy was observed between 70 and 100 kHz, at about 10 dB below the maximum peak in the spectrum (Fig. 7I).

### Wing resonances

**Figure 8:**
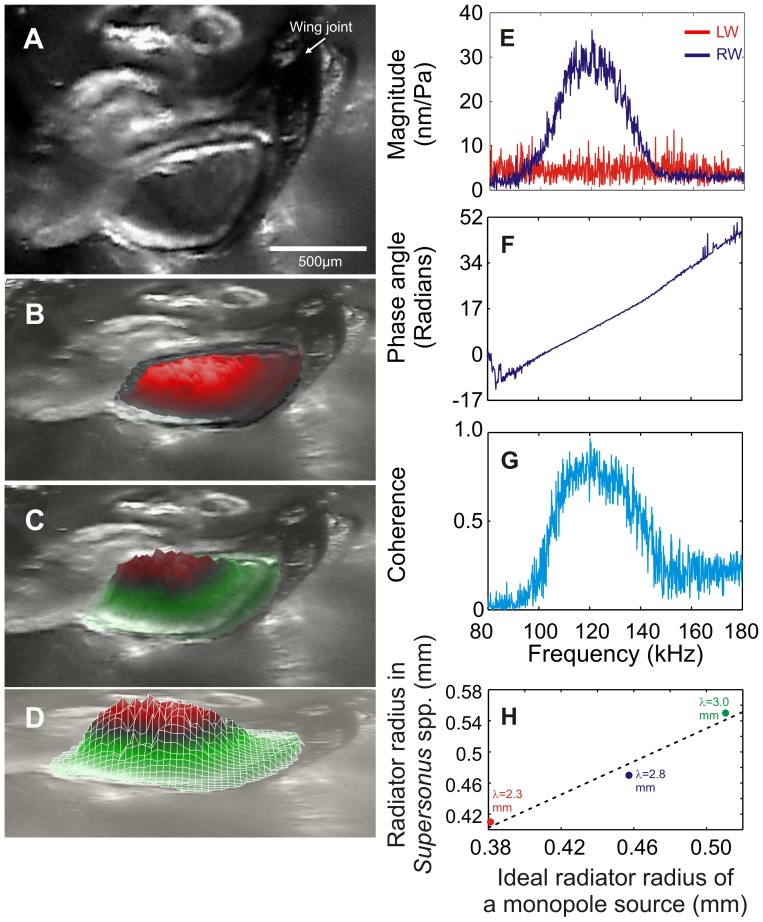
Wing resonance in *S. piercei* as measured with Laser Doppler Vibrometry. (**A–D**) Scanned area and defection shapes of the right wing (RW). A and B show the orientation image relating wing topology to the position of the scanning latice. (**C**, **D**) Area scans of mirror deflection at best response (122 kHz in this species). Wings scanned in normal position close to the body. Note how the mirror membrane strongly deflects while the rest of the wing veins and folded membranes rest in position. (**E**) Displacement and resonances of the left wing (LW) and RW. (**F**) Phase gain response of RW vibration. (**G**) Coherence across the frequency range measured for the RW response. (**H**) Expected and observed radiator size optimal for the frequencies used by *Supersonus* spp.

Wing resonance was measured from two species, *S. piercei* and *S. aequoreus*. Scanning laser vibrometry indicated the right-wing of *S. piercei* resonated at 122.4±1.7 kHz (Fig. 8). This resonance is close to the calling-song carrier (124 kHz). Q_−3dB_ for this observed wing resonance was 6.2±0.4 (n = 130 points). The left stridulatory wing did not show any deflection pattern, and no particularly sharp resonance was observed, at least in the frequency range measured (Fig. 8).

In *S. aequoreus* the right wing resonated at 155±6 kHz, (n = 2 specimens). This observed wing resonance is close to the dominant carrier of the calling-song (148 kHz, Fig. 7). Q_−3dB_ for this observed wing resonance was 9.2±1.1 (n = 220 scanning points). As in *S. piercei*, the left stridulatory wing did not show any deflection pattern in the range of frequencies used and does not show a particular sharp resonance.

## Discussion

*Supersonus* is a new genus of neotropical predaceous katydids with at least three species that use extraordinarily high ultrasonic frequencies (>115 kHz) for conspecific communication. Males produce these high-frequency mating calls with unusually high SPL. Typical SPL measures of katydids for microphones positioned at 10–15 cm dorsal to a specimen were 70–100 dB [12], [23], [26] though a few katydid species have been reported to exceed such output ranges (Table S1). For *Supersonus* spp., we hypothesize their unusual intensity to be the result of special features integrated into the mechanism of stridulation evolved by these insects: a monopole-like source, wing resonance, and wing deformation occurring under high shear forces. A monopole source radiates equally in all directions. By contrast, a dipole (doublet source) is like two monopoles of equal strength and opposite phase set back to back, alternately radiating sound with maxima normal to one side minima normal to the other [27], [28].

The wings of most male crickets and katydids are approximate examples of dipole sources [27]. In these insects, as the mirror and harp radiate sound from both upper and lower surfaces, moving with opposite phase they can experience destructive interference (short-circuiting) at their margins [27], [29]–[31]. For most katydid species such interference is mitigated because the forewings are enclosed laterally by a costal field (‘skirt’). This costal baffle, especially in species that are flightless and so are more free to evolve specialized baffles, plays a significant role to minimize short-circuiting between the two sides of the dipole. Our data suggest that *Supersonus* spp. may have adopted a wing structure and wing motion mechanics transforming this plesiomorphic dipole source into something approaching a monopole radiator.

The specific acoustic resistance and the efficiency of a sound source depend on the ratio between the source diameter and the sound wavelength [27]. In theory, a monopole source should have a minimum radius of 1/6 wavelength, a dipole a radius of 1/4 wavelength for good efficiency [27]. The 150 kHz produced by, for example, the call of *S. aequoreus* has a wavelength of ca 2.3 mm. For generation of such a wavelength, an efficient sound radiator (mirror and cavity beneath) should have a minimum radius of ca 0.38 mm as a monopole, 0.58 mm as a dipole. The mirror of *S. aequoreus* (the smallest of the 3 species in the genus, Fig. 2D) has a radius of ca 0.41 mm, and the height of the space below the mirror is between 0.2 and 0.3 mm. This suggests that the mechanism used by this species more closely approaches a monopole radiator than a dipole. Optimal and observed sound source dimensions for all three species are shown in Fig. 8H.

Protruding and partially isolated from the rest of the wing due to the smaller circumference of the ventral massive vein, the mirror of these *Supersonus* spp. is peculiar in functioning as a single vibrating disk backed by a well-enclosed cavity (Figs. 2 & 3). This design makes the right wing a closed box that radiates sound mostly through its dorsal surface (Fig. 8D). Different from most katydids, the wings of *Supersonus* spp. are not tilted at an angle over the notum during sound production. Rather the open side of the concave right wing is maintained in close contact with and parallel to the insect notum surface (see Video S1). The notum surface, the elevated mirror frame, its encircling veins and the adjoining wing tissue (Fig. 3), together create the enclosed box that we suggest produces the effect of a monopole-like radiator.

The carrier frequency of *Supersonus* spp. can be approximated by a mathematical model of the frequency of a membrane backed by a cavity:
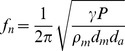
where, γ is the adiabatic constant of air, *P* is the pressure in the cavity, ρ_m_ is the density of the membrane, *d_m_* is the thickness of the membrane, and *d_a_* is the thickness (depth) of the air cavity [32]. From measurements of *S. piercei* (Fig 3) the height of the cavity is 0.21 mm, the membrane thickness is 1.18 µm, and membrane density (insect cuticle) is ca 1 kg/m^−3^ [33]). This general equation for a closed cavity with a membrane gives a resonant frequency of 120.4 kHz, a value very close to that of the calling song carrier (124 kHz for *S. piercei*) and that obtained from the resonant frequency of the wing (Fig. 8).

The observed high SPLs in *Supersonus* spp. can thus be explained by a combination of several variables. Mirror design and stridulating motion in *Supersonus* spp. could potentially optimize sound emission by approaching a monopole source, where monopoles are more efficient radiators than dipole sources [28], [34]. We show here that the right wing exhibits a relatively sharp resonance at specific frequencies (Fig. 8) and that this resonant frequency is stimulated by elevated tooth strike rates resulting from scraper elasticity [20]. The observed SPL results from the interaction of these variables: a monopole-like radiator, powered tooth impacts driven by scraper elasticity, and wing resonance (but see Montealegre-Z et al. [20]).

The elevated and projected mirror sits on a ventral ring formed by the massive vein that entirely encircles the ventral aspect of the wing (Fig. 3). The mirror frame is separated from this ring by softer tissue (atrophied cells and veins) that once had a role in sound radiation, e.g. the harp (Fig. 3). This softer tissue may work as a speaker surround during sound radiation. In human-made speakers the surround can be found around the perimeter of the cone. It is often made of foam, rubber, or other elastic materials [35]. The speaker surround serves two purposes: 1) it supplies the returning spring force necessary for the speaker to be a harmonic oscillator, and 2) it also aligns the cone correctly in the basket, which entails keeping the voice coil correctly aligned in the gap—the optimal area in the permanent magnetic field where the coil is designed to sit. These analogies therefore make the sound radiator in *Supersonus* species an interesting model for further studies of small resonators and speakers.

Other loud insects have been reported in nature. The cicada *Cyclochila australasiae* [36], the bladder grasshopper *Bullacris membracioides* [37], the water boatman bug *Micronecta scholtzi* [38], and the katydid *Panacanthus intensus* [26], are among the loudest insects known. The mechanism of the cicada and the bladder grasshopper can be roughly modelled, regarded as a pulsating sphere and so a nearly ideal monopole radiator. The mechanism of the water boatman bug involves a combination of tiny stridulatory structures and an air bubble resonator acting to propagate sound in water [39]. And the mechanism of *P. intensus* involves wing forces and perhaps wing deformation as well. However, all these insects operate in the audio range. Loudness at extreme ultrasonic frequencies is not that common and has been only reported in *Arachnoscelis arachnoides* singing at 74 kHz [40]. Since ultrasound suffers excess attenuation in air, animals need mechanisms to increase amplitude in order to maximize range, although high intensities might function to impress a female in terms of male quality.

The observed low wing speeds in both species recorded with HSV suggest the scraper is moving faster than the wings at closing to provide an elevated tooth strike rate. Such scraper speed, uncoupled from wing closure and involving scraper deformation, could be the result of stored elastic energy as proposed by Montealegre-Z et al. [20]. Several other species singing above 40 kHz seem to utilise such a mechanism [9], [20], including a newly reported high-ultrasonic Phaneropterinae katydid [41].

## Systematics and Taxonomy

A number of species, including those dealt with in the present paper, were erroneously assigned to the genus *Arachnoscelis* [42]–[46], mainly because of their shared general appearance, i.e. long and spiny legged and resembling spiders (Fig. 1). Recently Montealegre-Z et al. [21] redescribed the genus *Arachnoscelis*, from a particularly abundant population of *Arachnoscelis arachnoides*, the type species of the genus as originally described by Redtenbacher 1891 [47]. Those authors highlighted the fact that *A. arachnoides* does not share immediate synapomorphic features with the other species (including some *Supersonus* spp.) misdescribed within *Arachoscelis* after the original description by Karny 1911 [48].

Gorochov [49] created the subtribe Arachnoscelidina within the Meconematinae to incorporate two subgenera *Centrophisis* and *Peruphysis*, within *Arachnoscelis s. str*. Gorochov's delimitation of these two subgenera was made primarily on the male terminalia. However, it is difficult to assign *Supersonus* to either of these groups following the descriptions of the author of the genitalia (unfortunately the article lacks illustrations). In a strict molecular analysis of the Tettigoniidae, Muglestone et al. [13] found that the position of the Meconematinae and Listroscelidinae was not well resolved. These subfamilies were recovered as paraphyletic in their analysis. We place this new genus within the Listroscelidinae until new molecular analyses, at higher resolution, can illuminate the position of these groups. In this paper we present a combination of morphological, biophysical, and behavioural characters that we believe better help to distinguish this *Supersonus* from *Arachnoscelis*.

*Supersonus* differs from *Arachnoscelis* in the shape of the male's head, male terminalia, and tegminal venation. Adult males of *Arachnoscelis* possess a large head with developed mandibles [21], while the head and mandibles are not specialized in *Supersonus*. In *Arachnoscelis*, the male cerci are elongated and incurved, and the titillators are longer and sickle-shaped.

A major difference between *Arachnoscelis* and *Supersonus* was found in the male 10^th^ tergite. In *Arachnoscelis* this sclerite ends in a pair of rather small lateral lobes. The male genital (subgenital) plate has a pair of posterolateral lobes with a wide median notch between them. In *Arachnoscelis*, the left tegmen preserves a reduced mirror and harp cell, while in *Supersonus* the left wing is acoustically damped (Fig. 8E) and no membranous cells are present (Fig. 2A, 2B & 2C). In *Arachnoscelis*, the right wing has a functional mirror and harp cell, but in *Supersonus* the harp is absent and only the mirror is preserved as a sound radiating structure.

In *Arachnoscelis*, a massive vein (formed by the merging of several veins) curves towards the anal area, partially encircles the wing but ends bluntly leaving a soft flexible area (Fig. 2D). This massive vein weakly connects to CuPaa2 through the narrow handle vein (h in Fig. 2G) and vein CuPaa2+CuPaβ. In *Supersonus* the mirror frame preserves connection with this massive vein through veins h and CuPaa2+CuPaβ as in *Arachnoscelis* (Fig. 2D). However, in contrast to *Arachnoscelis*, the massive vein encircles the entire wing, forming a ring that is smaller than the mirror frame (see Fig. S3). This ring appears to constrict the membranes and veins adjacent to the mirror as if it were forcing the mirror frame to protrude upwards (Figs. 2D, 2E, and 2F). Therefore the mirror of *Supersonus* spp. is situated atop a concave cavity formed by a basal ring (the massive vein circle) and folded wing cells, and radiates a pure signal using a very tight baffling system.

Finally, there are strong behavioural differences in sound production between the two genera. *Arachnoscelis arachnoides* produce their calls at ca 74 kHz during the opening phase of the wings [40], *Supersonus* males operate as in most katydids, during the closing phase, reaching their more extreme ultrasonic levels (>120 kHz).

## Supporting Information

Figure S1 **External morphology of the left tympanal slits in** ***Supersonus*** **spp. (A–C)** Males. (**D–F**) Females.
(JPG)

Figure S2 **External morphology of the egg in** ***Supersonus*** **spp.** (**A–C**). Lateral view. (**D–F**). Top view of the anterior end showing the micropyle.
(JPG)

Figure S3 **Comparative anatomy of the wings of** ***Supersonus*** **and** ***Arachnoscelis*** **.** (**A**) The wings of *S. piercei* and *A. arachnoides* under same scale. (**B**) Wing venation patterns in both species. Wings have been magnified to a similar size for comparative purposes.
(JPG)

Table S1 Submitted as a pdf file with information an relevant references inserted in the same document.
(PDF)

Video S1 **High-speed video recording of** ***Supersonus aequoreus*** **during sound production.** Video recorded at 1000 fps, sound sampled at 400 k-samples/s, and slowed down 100x, sound resampled at 48 k-samples/s.
(MP4)

Video S2 **High-speed video recording of** ***Supersonus piercei*** **during sound production.** Video recorded at 1000 fps, sound sampled at 300 k-samples/s, and slowed down 110x, sound resampled at 48 k-samples/s.
(MP4)
