# Supplementary figures and images for: Shrinking Wings for Ultrasonic Pitch Production: Hyperintense Ultra-Short-Wavelength Calls in a New Genus of Neotropical Katydids (Orthoptera: Tettigoniidae)
Source: PLoS One. 2014 Jun 5;9(6):e98708. doi: 10.1371/journal.pone.0098708 (PMC4047022; doi:10.1371/journal.pone.0098708)

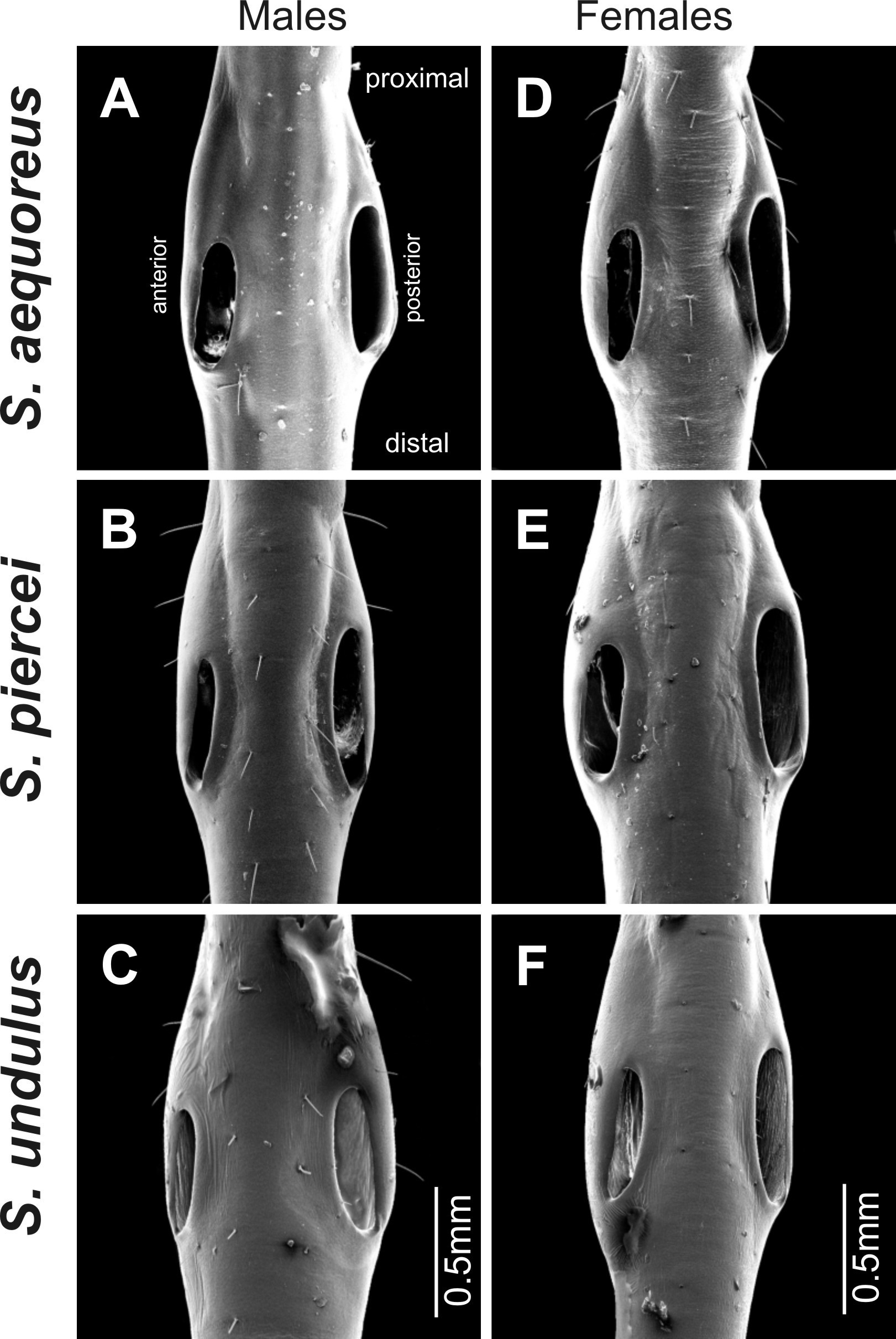

Supplement: Figure S1 — External morphology of the left tympanal slits in Supersonus spp. (A–C) Males. (D–F) Females. (JPG) [file pone.0098708.s001.jpg]

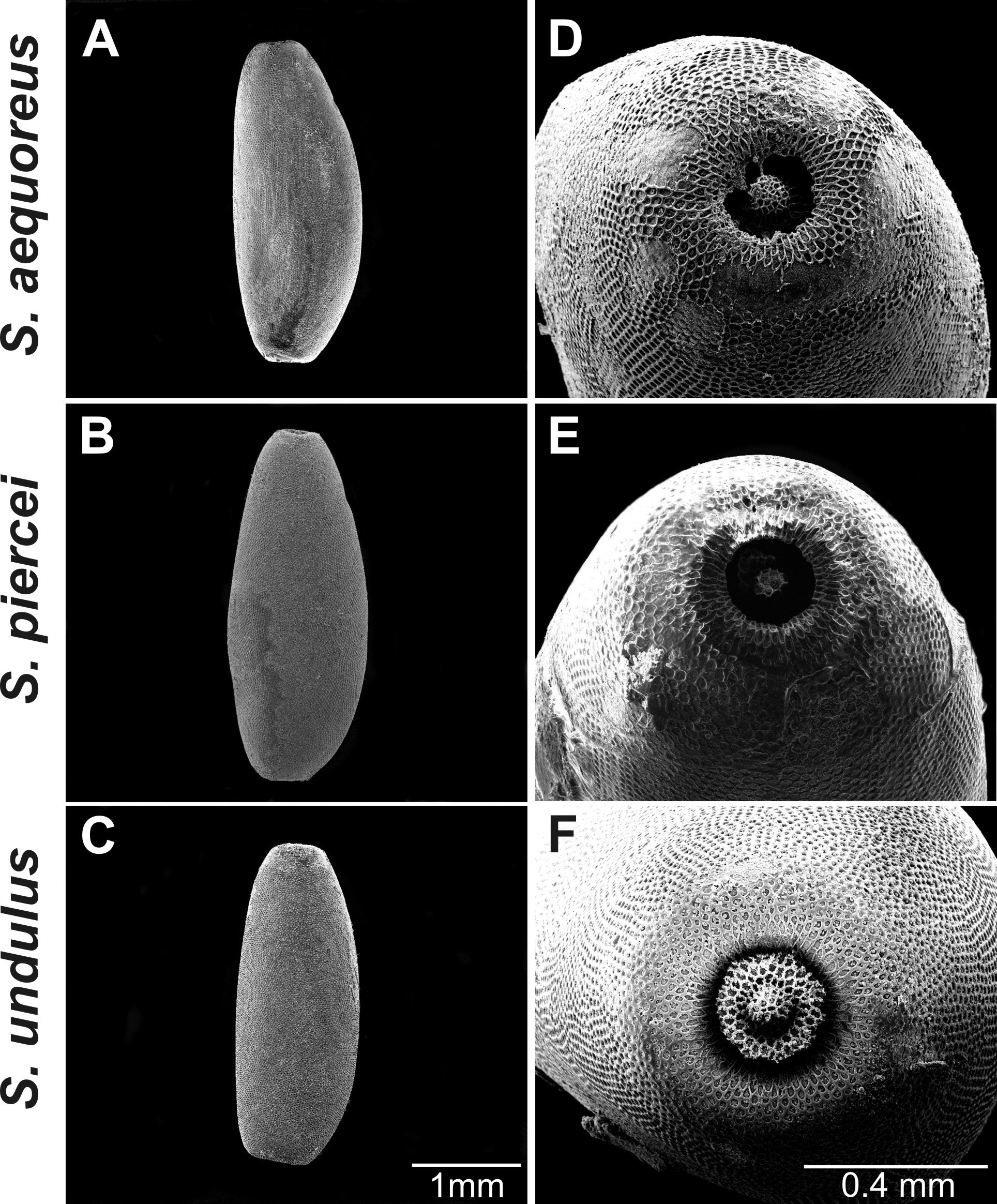

Supplement: Figure S2 — External morphology of the egg in Supersonus spp. (A–C). Lateral view. (D–F). Top view of the anterior end showing the micropyle. (JPG) [file pone.0098708.s002.jpg]

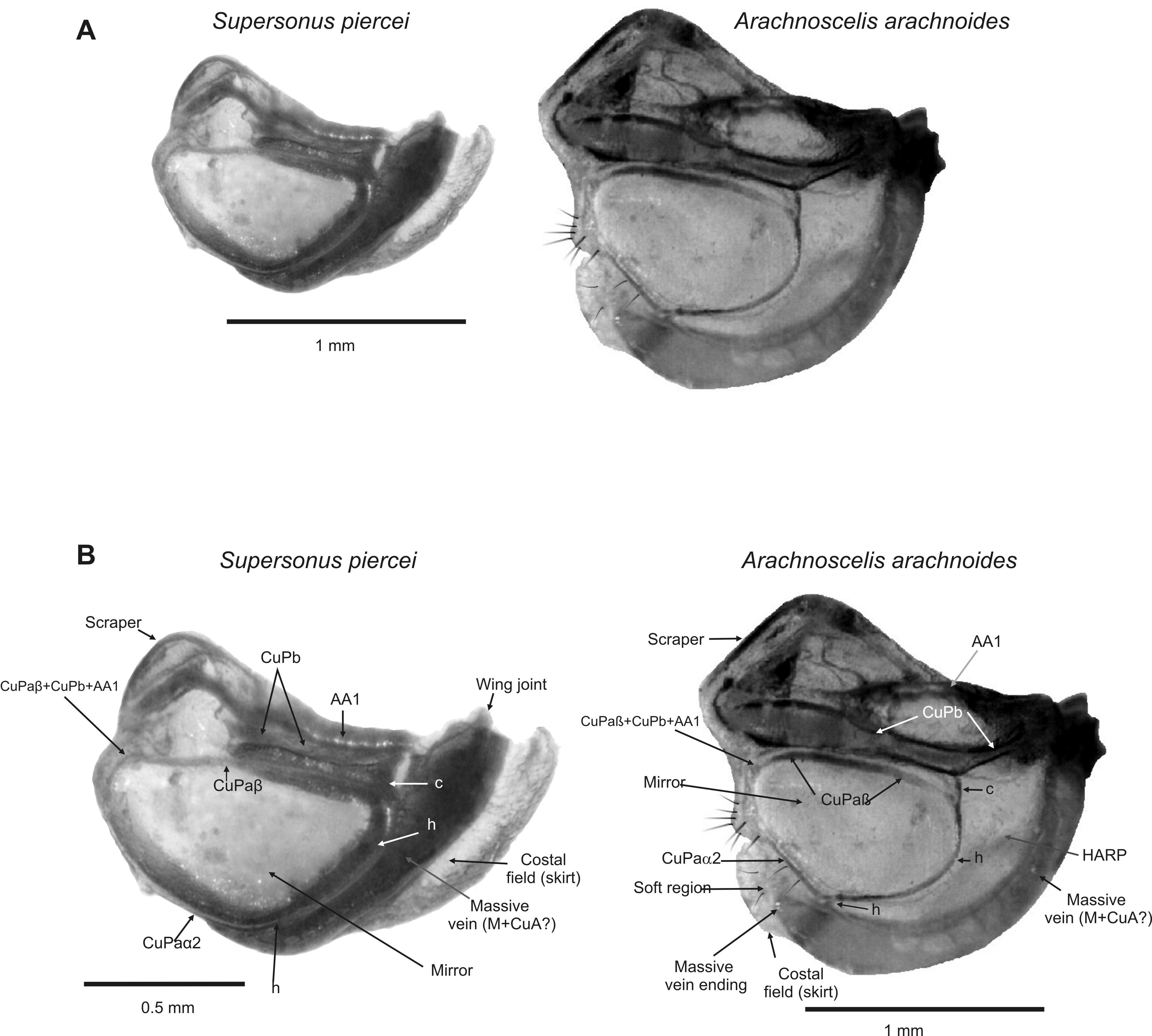

Supplement: Figure S3 — Comparative anatomy of the wings of Supersonus and Arachnoscelis . (A) The wings of S. piercei and A. arachnoides under same scale. (B) Wing venation patterns in both species. Wings have been magnified to a similar size for comparative purposes. (JPG) [file pone.0098708.s003.jpg]
