# Supplementary material for: Shrinking Wings for Ultrasonic Pitch Production: Hyperintense Ultra-Short-Wavelength Calls in a New Genus of Neotropical Katydids (Orthoptera: Tettigoniidae)
Source: PLoS One. 2014 Jun 5;9(6):e98708. doi: 10.1371/journal.pone.0098708 (PMC4047022; doi:10.1371/journal.pone.0098708)
Supplement: Table S1 — Submitted as a pdf file with information an relevant references inserted in the same document. (PDF) [file pone.0098708.s004.pdf]

Table S1. Carrier frequencies (f_c), sound pressure levels (SPL) of some Tettigoniidae species, and microphone distance from the specimen.

Species	f_c (kHz)	SPL (dB)	Microphone distance (cm)	Source
Panacanthus pallicornis	4.7	97.6	10	Montealegre-Z & Morris 2004 [1]
Panacanthus gibbosus	6.4	95	10	Montealegre-Z & Morris 2004 [1]
Copiphora rhinoceros	8.7	94	10	Morris 1980 [2]
Typophyllum mortuifolium	9.4	93	7	Morris et al. 1989 [3]
Panacanthus intensus	11.7	113	10	Montealegre-Z & Morris 2004 [1]
Poecilimon affinis	13	112	10	Heller & von Helversen, 1993 [4]
Typophyllum bolivary	14	83	10	Morris et al. 1989 [3]
Choeroparnops gigliotosi	15	102	10	Morris et al. 1994 [5]
Isophya taurica	15	105	10	Heller 1988 [6]
Isophya rossica	15	106	10	Heller 1988 [6]
Decticus verrucivorus	15	110	10	Heller 1988 [6]
Isophya stysi	18	100	10	Heller 1988 [6]
Isophya stepposa	19	92	10	Heller 1988 [6]
Metrioptera bicolor	20	105	10	Heller 1988 [6]
Platycleis intermedia	22	101	3	Heller 1988 [6]
Docidocercus gigliotosi	23	94	10	Morris et al. 1994 [5]
Docidocercus chlorops	24	104	9	Morris et al. 1989 [3]
Isophya sp.	25	95	10	Heller 1988 [6]
Isophya brunneri	25	125	10	Heller 1988 [6]
Metrioptera roeselii	25	105	10	Heller 1988 [6]
Metrioptera brachyptera	30	110	10	Heller 1988 [6]
Copiphora brevirostris	31.3	95	30	Morris et al. 1994 [5]
Tettigonia cantans	35	100	10	Heller 1988 [6]
Kawanaphila yarraga	40.3	116.5	10	Mason & Bailey 1998 [7]
Kawanaphila mirla	70.5	114.9	10	Mason & Bailey 1998 [7]
Arachnoscelis	74	115	10	Chivers et al, 2013 [8]

arachnoides				
Mypophyllum speciosum	83.3	100.2	9.5	Morris et al. 1994 [5]
Ectomoptera nepicauda	100	unknown	unknown	Heller & Hemp, 2014 [9]
Haenschiella sp.	105.5	79.7	10	Morris et al. 1994 [5]
Supersonus undulus	115.2	113	15	this paper
Supersonus piercei	124.5	111	15	this paper
Supersonus aequoreus	148.3	115	15	this paper

References

1. Montealegre-Z F, Morris GK (2004) The spiny devil katydids, *Panacanthus* Walker (Orthoptera : Tettigoniidae): an evolutionary study of acoustic behaviour and morphological traits. Syst Entomol 29: 21-57.
2. Morris GK (1980) Calling display and mating behaviour of *Copiphora rhinoceros* Pictet (Orthoptera, Tettigoniidae). Anim Behav 28: 42-51.
3. Morris GK, Klimas DE, Nickle DA (1989) Acoustic signals and systematics of false-leaf Katydids from Ecuador (Orthoptera, Tettigoniidae, Pseudophyllinae). Trans Am Entomol Soc (Phila) 114: 215-263.
4. Heller K-G, Helversen D (1993) Calling behavior in bushcrickets of the genus Poecilimon with differing communication systems (Orthoptera: Tettigonioidea, Phaneropteridae). J Insect Behav 6: 361-377.
5. Morris GK, Mason AC, Wall P, Belwood JJ (1994) High ultrasonic and tremulation signals in neotropical katydids (Orthoptera, Tettigoniidae). J Zool (Lond) 233: 129-163.
6. Heller KG (1988) Bioakustik der Europäischen Laubheuschrecken. Weikersheim: Verlag Josef Margraf.
7. Mason AC, Bailey WJ (1998) Ultrasound hearing and male-male communication in Australian katydids (Tettigoniidae : Zaprochilinae) with sexually dimorphic ears. Physiol Entomol 23: 139-149.
8. Chivers B, Jonsson T, Cadena-Castaneda OJ, Montealegre-Z F (2013) Ultrasonic reverse stridulation in the spider-like katydid *Arachnoscelis* (Orthoptera: Listrosceledinae). Bioacoustics 23: 67–77.
9. Heller KG, Hemp C (2014) Fiddler on the Tree - A Bush-Cricket Species with Unusual Stridulatory Organs and Song. PloS ONE 9: e92366.
